# (*E*)-1,1′-Dibutyl-3,3′-biindolinyl­idene-2,2′-dione

**DOI:** 10.1107/S160053681005066X

**Published:** 2010-12-08

**Authors:** Mao-Sen Yuan, Qi Fang

**Affiliations:** a College of Science, Northwest Sci-Tech University of Agriculture and Forestry, Yangling 712100, Shanxi Province, People’s Republic of China; b State Key Laboratory of Crystal Materials, Shandong University, Jinan 250100, Shandong Province, People’s Republic of China

## Abstract

In the title mol­ecule, C_24_H_26_N_2_O_2_, the two indol-2-one units, which are connected by a C=C double bond, are almost coplanar with an inter­planar angle of 6.8 (1)°. On cooling from 293 to 120 K, the space group changes from *P*2_1_/*n* to *P*2_1_. Two intra­molecular C—H⋯O hydrogen bonds occur.

## Related literature

For uses of isoindigo derivatives as medicines, see: Sassatelli *et al.* (2004 ▶). For the room temperature (293 K) structure, see: Yuan *et al.* (2007 ▶).
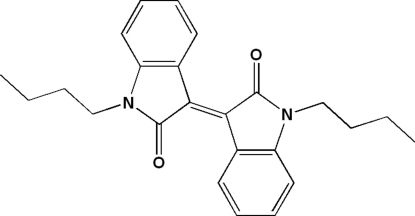


## Experimental

### 

#### Crystal data

C_24_H_26_N_2_O_2_
*M* *_r_* = 374.47
Monoclinic, 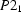

*a* = 8.9224 (3) Å
*b* = 11.9605 (5) Å
*c* = 9.6827 (4) Å
β = 110.782 (1)°
*V* = 966.07 (7) Å^3^
*Z* = 2
Mo *K*α radiation
μ = 0.08 mm^−1^
*T* = 120 K
0.20 × 0.11 × 0.09 mm

#### Data collection

Bruker SMART 6K CCD area-detector diffractometer
Absorption correction: multi-scan (*SADABS*; Bruker, 2006 ▶) *T*_min_ = 0.984, *T*_max_ = 0.993
13014 measured reflections
2926 independent reflections
2477 reflections with *I* > 2σ(*I*)
*R*_int_ = 0.041

#### Refinement

*R*[*F* ^2^ > 2σ(*F* ^2^)] = 0.044
*wR*(*F* ^2^) = 0.121
*S* = 1.04
2926 reflections
257 parameters
1 restraint
H-atom parameters constrained
Δρ_max_ = 0.38 e Å^−3^
Δρ_min_ = −0.22 e Å^−3^

### 

Data collection: *SMART* (Bruker, 2001 ▶); cell refinement: *SAINT* (Bruker, 2001 ▶); data reduction: *SAINT*; program(s) used to solve structure: *SHELXTL* (Sheldrick, 2008 ▶); program(s) used to refine structure: *SHELXTL*; molecular graphics: *SHELXTL*; software used to prepare material for publication: *SHELXTL*.

## Supplementary Material

Crystal structure: contains datablocks I, global. DOI: 10.1107/S160053681005066X/jh2237sup1.cif

Structure factors: contains datablocks I. DOI: 10.1107/S160053681005066X/jh2237Isup2.hkl

Additional supplementary materials:  crystallographic information; 3D view; checkCIF report

## Figures and Tables

**Table 1:** Hydrogen-bond geometry (Å, °)

*D*—H⋯*A*	*D*—H	H⋯*A*	*D*⋯*A*	*D*—H⋯*A*
C4—H4⋯O2	0.95	2.05	2.815 (3)	137
C24—H24⋯O1	0.95	2.04	2.805 (3)	136

**Fig. 1.**
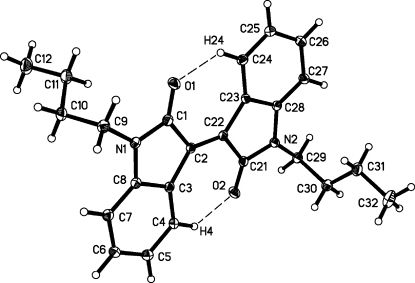
The molecular structure of (I). Displacement ellipsoids are drawn at the 30% probability level.

**Fig. 2.**
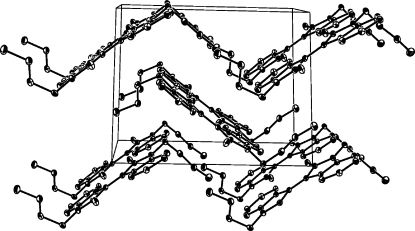
An a axis view of the molecular packing of (I) at 120 K.

## supplementary crystallographic information

### Comment

Isoindigo can be obtained from various natural sources. Its derivatives are usually known as useful medicine (Sassatelli *et al.*, 2004). Recently we have synthesized the title isoindigo derivative and determined its structure at room temperature (293 K) (Yuan *et al.*, 2007). To reduce the disorder of the two butyl groups of the molecule at room temperature, we redetermined the structure at low temperature (120 K). Unexpectedly, we found some changes of the crystal at different temperatures. Here we mainly report the correlation between structures at high and low temperatures.

The band features of the molecule at 120 K are very similar to those at 293 k. For example, the C3—C2═C22—C23 fragment is quite conjugated and exhibits an E configuration, in which the bond lengths are 1.479 (3), 1.373 (3), and 1.477 (3) for C3—C2, C2═C22, and C22—C23, respectively. There are a pair of intramolecular hydrogen bonds C4—H4···O2 and C24—H24···O1 (see Fig. 1).

The cell parameters of the compound at 293 K and 120 K are very close. The cell volumn 1005.32 (4) Å^3^ at 293 K slightly shrinks to 966.07 (7) Å^3^ at 120 K. The temperature effect on the molecule is significant that the severely disordered terminal C atoms of the two butyl groups at 293 K become completely ordered at 120 k and all the atomic displacement parameters are greatly reduced.

At 293 K, the molecule is centrosymmetric and has a perfect planarity. At 120 K, however, the centrosymmetry is borken and a dihedral angle of 6.8 (1) ° between the two nine-membered indole planes are developed. Consequently, molecular chirality is produced, which brings the chirality to the crystal. Meanwhile, the space group of the crystal changed from *P*2_1_/*n* to *P*2_1_.

### Experimental

1-Butyl-1*H*-indole-2,3-dione (1.5 g) and 1-butyl-1*H* -indole-2-one (1.5 g) were mixed with polyphosphoric acid (15 g), reacted at 333–338 K for 30 min. under N_2_, and then to 433–442 K with stirring. After 3 h, the mixture was poured into ice water and stirred for 1 h. The solution was extracted in chloroform and dried over Na_2_SO_4_. After removing the solvent, the crude product was purified by column chromatography on silica gel, eluting with petrol ether, affording the title compound (1.4 g, 47.1%). The compound was dissolved in THF and purple plate title crystals formed on slow evaporation at room temperature.

### Refinement

All H atoms were positioned geometrically and allowed to ride on their attached atom. The C—H bond lengths for aromatic, methyl and methene groups were set to 0.95, 0.98 and 0.99 Å, respectively.

### Crystal data

**Table:**

C_24_H_26_N_2_O_2_	*F*(000) = 400
*M**_r_* = 374.47	*D*_x_ = 1.287 Mg m^−^^3^
Monoclinic, *P*2_1_	Mo *K*α radiation, λ = 0.71073 Å
Hall symbol: P 2yb	Cell parameters from 3669 reflections
*a* = 8.9224 (3) Å	θ = 2.7–30.0°
*b* = 11.9605 (5) Å	µ = 0.08 mm^−^^1^
*c* = 9.6827 (4) Å	*T* = 120 K
β = 110.782 (1)°	Plank, purple
*V* = 966.07 (7) Å^3^	0.20 × 0.11 × 0.09 mm
*Z* = 2	

### Data collection

**Table:**

Bruker SMART 6K CCD area-detector diffractometer	2926 independent reflections
Radiation source: fine-focus sealed tube	2477 reflections with *I* > 2σ(*I*)
graphite	*R*_int_ = 0.041
Detector resolution: ω pixels mm^-1^	θ_max_ = 30.0°, θ_min_ = 2.3°
φ and ω scans	*h* = −12→12
Absorption correction: multi-scan (*SADABS*; Bruker, 2006)	*k* = −16→16
*T*_min_ = 0.984, *T*_max_ = 0.993	*l* = −13→13
13014 measured reflections	

### Refinement

**Table:**

Refinement on *F*^2^	Primary atom site location: structure-invariant direct methods
Least-squares matrix: full	Secondary atom site location: difference Fourier map
*R*[*F*^2^ > 2σ(*F*^2^)] = 0.044	Hydrogen site location: inferred from neighbouring sites
*wR*(*F*^2^) = 0.121	H-atom parameters constrained
*S* = 1.04	*w* = 1/[σ^2^(*F*_o_^2^) + (0.0708*P*)^2^ + 0.1553*P*] where *P* = (*F*_o_^2^ + 2*F*_c_^2^)/3
2926 reflections	(Δ/σ)_max_ = 0.003
257 parameters	Δρ_max_ = 0.38 e Å^−^^3^
1 restraint	Δρ_min_ = −0.22 e Å^−^^3^

### Special details

**Table:**

Experimental. The data collection nominally covered full sphere of reciprocal space, by a combination of 3 runs of narrow-frame ω-scans (scan width 0.3° ω, 20 s exposure), every run at a different φ angle. Crystal to detector distance 4.83 cm.
Geometry. All e.s.d.'s (except the e.s.d. in the dihedral angle between two l.s. planes)are estimated using the full covariance matrix. The cell e.s.d.'s are takeninto account individually in the estimation of e.s.d.'s in distances, anglesand torsion angles; correlations between e.s.d.'s in cell parameters are onlyused when they are defined by crystal symmetry. An approximate (isotropic)treatment of cell e.s.d.'s is used for estimating e.s.d.'s involving l.s. planes.
Refinement. Refinement of *F*^2^ against ALL reflections. The weighted *R*-factor *wR* and goodness of fit *S* are based on *F*^2^, conventional *R*-factors *R* are based on *F*, with *F* set to zero for negative *F*^2^. The threshold expression of *F*^2^ > σ(*F*^2^) is used only for calculating *R*-factors(gt) *etc*. and is not relevant to the choice of reflections for refinement. *R*-factors based on *F*^2^ are statistically about twice as large as those based on *F*, and *R*- factors based on ALL data will be even larger. Methyl groups were refined as rigid bodies rotating around C—C bonds, with a common refined U for three H atoms. Other H atoms: riding model.

### Fractional atomic coordinates and isotropic or equivalent isotropic displacement parameters (Å^2^)

**Table:**

	*x*	*y*	*z*	*U*_iso_*/*U*_eq_	
O1	0.5183 (2)	0.40482 (16)	0.3409 (2)	0.0316 (4)	
O2	0.9572 (2)	0.57182 (16)	0.1333 (2)	0.0298 (4)	
N1	0.7459 (2)	0.30308 (16)	0.4455 (2)	0.0223 (4)	
N2	0.7454 (2)	0.69090 (16)	0.0617 (2)	0.0212 (4)	
C1	0.6589 (3)	0.3867 (2)	0.3584 (2)	0.0219 (4)	
C2	0.7669 (2)	0.44723 (18)	0.2919 (2)	0.0192 (4)	
C3	0.9220 (2)	0.38775 (19)	0.3518 (2)	0.0195 (4)	
C4	1.0726 (3)	0.3982 (2)	0.3403 (3)	0.0227 (4)	
H4	1.0912	0.4548	0.2795	0.027*	
C5	1.1959 (3)	0.3257 (2)	0.4182 (3)	0.0266 (5)	
H5	1.2985	0.3338	0.4103	0.032*	
C6	1.1715 (3)	0.2420 (2)	0.5069 (3)	0.0265 (5)	
H6A	1.2570	0.1932	0.5587	0.032*	
C7	1.0227 (3)	0.2291 (2)	0.5203 (3)	0.0257 (5)	
H7	1.0049	0.1719	0.5809	0.031*	
C8	0.9016 (3)	0.30130 (19)	0.4437 (2)	0.0210 (4)	
C9	0.6854 (3)	0.2281 (2)	0.5331 (2)	0.0259 (5)	
H9A	0.5909	0.2631	0.5466	0.031*	
H9B	0.7690	0.2188	0.6321	0.031*	
C10	0.6377 (3)	0.1131 (2)	0.4634 (3)	0.0289 (5)	
H10A	0.7291	0.0810	0.4414	0.035*	
H10B	0.6157	0.0633	0.5356	0.035*	
C11	0.4915 (3)	0.1152 (2)	0.3223 (3)	0.0330 (5)	
H11A	0.4000	0.1472	0.3440	0.040*	
H11B	0.5135	0.1646	0.2496	0.040*	
C12	0.4457 (4)	0.0003 (2)	0.2548 (3)	0.0424 (7)	
H12A	0.3545	0.0068	0.1617	0.049 (6)*	
H12B	0.4161	−0.0474	0.3233	0.049 (6)*	
H12C	0.5370	−0.0328	0.2358	0.049 (6)*	
C21	0.8248 (2)	0.59781 (19)	0.1318 (2)	0.0205 (4)	
C22	0.7168 (2)	0.53754 (18)	0.1994 (2)	0.0193 (4)	
C23	0.5658 (2)	0.60189 (19)	0.1468 (2)	0.0186 (4)	
C24	0.4134 (2)	0.5911 (2)	0.1555 (2)	0.0230 (4)	
H24	0.3907	0.5296	0.2070	0.028*	
C25	0.2947 (3)	0.6699 (2)	0.0894 (3)	0.0243 (5)	
H25	0.1908	0.6605	0.0940	0.029*	
C26	0.3261 (3)	0.7618 (2)	0.0169 (2)	0.0253 (5)	
H26	0.2445	0.8158	−0.0254	0.030*	
C27	0.4764 (3)	0.7760 (2)	0.0054 (3)	0.0245 (5)	
H27	0.4993	0.8391	−0.0432	0.029*	
C28	0.5909 (2)	0.69476 (18)	0.0674 (2)	0.0199 (4)	
C29	0.8118 (3)	0.7658 (2)	−0.0215 (2)	0.0240 (4)	
H29A	0.7229	0.8062	−0.0964	0.029*	
H29B	0.8676	0.7207	−0.0742	0.029*	
C30	0.9289 (3)	0.8506 (2)	0.0767 (3)	0.0287 (5)	
H30A	1.0197	0.8098	0.1487	0.034*	
H30B	0.9724	0.8966	0.0146	0.034*	
C31	0.8570 (3)	0.9279 (2)	0.1606 (3)	0.0321 (5)	
H31A	0.8193	0.8831	0.2279	0.039*	
H31B	0.7631	0.9667	0.0898	0.039*	
C32	0.9782 (4)	1.0145 (3)	0.2504 (3)	0.0479 (8)	
H32A	1.0220	1.0551	0.1853	0.054 (6)*	
H32B	0.9249	1.0674	0.2953	0.054 (6)*	
H32C	1.0654	0.9769	0.3283	0.054 (6)*	

### Atomic displacement parameters (Å^2^)

**Table:**

	*U*^11^	*U*^22^	*U*^33^	*U*^12^	*U*^13^	*U*^23^
O1	0.0265 (8)	0.0276 (9)	0.0468 (11)	0.0064 (7)	0.0204 (8)	0.0120 (8)
O2	0.0262 (8)	0.0267 (9)	0.0415 (9)	0.0058 (7)	0.0183 (7)	0.0102 (7)
N1	0.0218 (8)	0.0200 (9)	0.0262 (9)	−0.0022 (7)	0.0100 (7)	0.0037 (7)
N2	0.0202 (8)	0.0200 (9)	0.0245 (9)	0.0009 (7)	0.0093 (7)	0.0034 (7)
C1	0.0236 (10)	0.0197 (10)	0.0245 (10)	−0.0014 (9)	0.0112 (8)	−0.0011 (8)
C2	0.0201 (9)	0.0160 (9)	0.0226 (10)	−0.0010 (8)	0.0091 (7)	−0.0018 (7)
C3	0.0207 (9)	0.0173 (10)	0.0195 (9)	−0.0012 (8)	0.0060 (7)	−0.0011 (8)
C4	0.0226 (9)	0.0181 (10)	0.0269 (10)	0.0000 (9)	0.0083 (8)	0.0018 (8)
C5	0.0216 (10)	0.0267 (12)	0.0308 (12)	−0.0002 (9)	0.0084 (9)	0.0022 (10)
C6	0.0222 (10)	0.0251 (11)	0.0284 (11)	0.0016 (9)	0.0042 (8)	0.0046 (9)
C7	0.0252 (11)	0.0226 (11)	0.0263 (11)	−0.0017 (9)	0.0054 (9)	0.0036 (9)
C8	0.0212 (9)	0.0190 (10)	0.0223 (10)	−0.0014 (8)	0.0071 (7)	−0.0003 (8)
C9	0.0282 (11)	0.0263 (11)	0.0243 (10)	−0.0022 (10)	0.0107 (9)	0.0059 (9)
C10	0.0314 (11)	0.0224 (11)	0.0345 (12)	0.0002 (10)	0.0138 (9)	0.0070 (9)
C11	0.0379 (13)	0.0232 (11)	0.0337 (12)	−0.0016 (10)	0.0077 (10)	0.0016 (10)
C12	0.0505 (17)	0.0257 (13)	0.0462 (16)	−0.0037 (12)	0.0113 (13)	−0.0035 (11)
C21	0.0221 (9)	0.0174 (10)	0.0228 (10)	−0.0009 (8)	0.0091 (8)	0.0011 (8)
C22	0.0207 (9)	0.0164 (9)	0.0215 (10)	−0.0019 (8)	0.0083 (7)	−0.0020 (7)
C23	0.0180 (8)	0.0163 (9)	0.0200 (9)	0.0012 (8)	0.0051 (7)	−0.0016 (7)
C24	0.0214 (10)	0.0237 (11)	0.0251 (10)	−0.0021 (9)	0.0097 (8)	0.0000 (8)
C25	0.0208 (10)	0.0249 (12)	0.0276 (11)	−0.0008 (9)	0.0091 (8)	−0.0003 (9)
C26	0.0232 (10)	0.0257 (11)	0.0261 (10)	0.0054 (9)	0.0076 (8)	0.0030 (9)
C27	0.0232 (10)	0.0225 (11)	0.0274 (10)	0.0025 (9)	0.0083 (8)	0.0047 (9)
C28	0.0198 (9)	0.0191 (10)	0.0205 (9)	−0.0008 (8)	0.0067 (7)	−0.0017 (8)
C29	0.0255 (10)	0.0215 (10)	0.0269 (11)	−0.0010 (9)	0.0117 (9)	0.0048 (9)
C30	0.0281 (11)	0.0249 (11)	0.0347 (12)	−0.0038 (9)	0.0129 (9)	0.0052 (9)
C31	0.0365 (12)	0.0227 (11)	0.0361 (13)	−0.0028 (10)	0.0114 (10)	0.0009 (9)
C32	0.064 (2)	0.0330 (15)	0.0429 (16)	−0.0158 (15)	0.0146 (14)	−0.0077 (12)

### Geometric parameters (Å, °)

**Table:**

O1—C1	1.224 (3)	C11—H11B	0.9900
O2—C21	1.217 (3)	C12—H12A	0.9801
N1—C1	1.360 (3)	C12—H12B	0.9801
N1—C8	1.396 (3)	C12—H12C	0.9801
N1—C9	1.463 (3)	C21—C22	1.524 (3)
N2—C21	1.364 (3)	C22—C23	1.477 (3)
N2—C28	1.399 (3)	C23—C24	1.398 (3)
N2—C29	1.464 (3)	C23—C28	1.413 (3)
C1—C2	1.519 (3)	C24—C25	1.392 (3)
C2—C22	1.373 (3)	C24—H24	0.9500
C2—C3	1.479 (3)	C25—C26	1.386 (3)
C3—C4	1.393 (3)	C25—H25	0.9500
C3—C8	1.417 (3)	C26—C27	1.394 (3)
C4—C5	1.394 (3)	C26—H26	0.9500
C4—H4	0.9500	C27—C28	1.383 (3)
C5—C6	1.386 (3)	C27—H27	0.9500
C5—H5	0.9500	C29—C30	1.523 (3)
C6—C7	1.388 (3)	C29—H29A	0.9900
C6—H6A	0.9500	C29—H29B	0.9900
C7—C8	1.376 (3)	C30—C31	1.514 (4)
C7—H7	0.9500	C30—H30A	0.9900
C9—C10	1.525 (3)	C30—H30B	0.9900
C9—H9A	0.9900	C31—C32	1.528 (4)
C9—H9B	0.9900	C31—H31A	0.9900
C10—C11	1.518 (3)	C31—H31B	0.9900
C10—H10A	0.9900	C32—H32A	0.9802
C10—H10B	0.9900	C32—H32B	0.9802
C11—C12	1.513 (4)	C32—H32C	0.9802
C11—H11A	0.9900		
			
C1—N1—C8	110.82 (18)	C11—C12—H12C	109.4
C1—N1—C9	124.27 (18)	H12A—C12—H12C	109.5
C8—N1—C9	124.88 (19)	H12B—C12—H12C	109.5
C21—N2—C28	110.65 (17)	O2—C21—N2	123.0 (2)
C21—N2—C29	122.21 (18)	O2—C21—C22	129.3 (2)
C28—N2—C29	126.74 (18)	N2—C21—C22	107.66 (17)
O1—C1—N1	123.2 (2)	C2—C22—C23	132.94 (19)
O1—C1—C2	129.1 (2)	C2—C22—C21	122.89 (18)
N1—C1—C2	107.73 (17)	C23—C22—C21	104.12 (18)
C22—C2—C3	132.62 (18)	C24—C23—C28	116.8 (2)
C22—C2—C1	122.87 (17)	C24—C23—C22	136.0 (2)
C3—C2—C1	104.50 (18)	C28—C23—C22	107.22 (18)
C4—C3—C8	117.3 (2)	C25—C24—C23	120.5 (2)
C4—C3—C2	136.1 (2)	C25—C24—H24	119.7
C8—C3—C2	106.67 (18)	C23—C24—H24	119.7
C3—C4—C5	120.0 (2)	C26—C25—C24	120.8 (2)
C3—C4—H4	120.0	C26—C25—H25	119.6
C5—C4—H4	120.0	C24—C25—H25	119.6
C6—C5—C4	121.2 (2)	C25—C26—C27	120.7 (2)
C6—C5—H5	119.4	C25—C26—H26	119.7
C4—C5—H5	119.4	C27—C26—H26	119.7
C5—C6—C7	120.3 (2)	C28—C27—C26	117.6 (2)
C5—C6—H6A	119.9	C28—C27—H27	121.2
C7—C6—H6A	119.9	C26—C27—H27	121.2
C8—C7—C6	118.3 (2)	C27—C28—N2	126.3 (2)
C8—C7—H7	120.9	C27—C28—C23	123.57 (19)
C6—C7—H7	120.9	N2—C28—C23	110.10 (18)
C7—C8—N1	126.7 (2)	N2—C29—C30	112.71 (18)
C7—C8—C3	123.1 (2)	N2—C29—H29A	109.0
N1—C8—C3	110.28 (19)	C30—C29—H29A	109.0
N1—C9—C10	113.50 (19)	N2—C29—H29B	109.0
N1—C9—H9A	108.9	C30—C29—H29B	109.0
C10—C9—H9A	108.9	H29A—C29—H29B	107.8
N1—C9—H9B	108.9	C31—C30—C29	114.5 (2)
C10—C9—H9B	108.9	C31—C30—H30A	108.6
H9A—C9—H9B	107.7	C29—C30—H30A	108.6
C11—C10—C9	113.5 (2)	C31—C30—H30B	108.6
C11—C10—H10A	108.9	C29—C30—H30B	108.6
C9—C10—H10A	108.9	H30A—C30—H30B	107.6
C11—C10—H10B	108.9	C30—C31—C32	111.7 (2)
C9—C10—H10B	108.9	C30—C31—H31A	109.3
H10A—C10—H10B	107.7	C32—C31—H31A	109.3
C12—C11—C10	112.8 (2)	C30—C31—H31B	109.3
C12—C11—H11A	109.0	C32—C31—H31B	109.3
C10—C11—H11A	109.0	H31A—C31—H31B	107.9
C12—C11—H11B	109.0	C31—C32—H32A	109.5
C10—C11—H11B	109.0	C31—C32—H32B	109.5
H11A—C11—H11B	107.8	H32A—C32—H32B	109.5
C11—C12—H12A	109.5	C31—C32—H32C	109.5
C11—C12—H12B	109.5	H32A—C32—H32C	109.5
H12A—C12—H12B	109.5	H32B—C32—H32C	109.5

### Hydrogen-bond geometry (Å, °)

**Table:**

*D*—H···*A*	*D*—H	H···*A*	*D*···*A*	*D*—H···*A*
C4—H4···O2	0.95	2.05	2.815 (3)	137
C24—H24···O1	0.95	2.04	2.805 (3)	136
